# Preparation and Characterization of Ni Spines Grown on the Surface of Cubic Boron Nitride Grains by Electroplating Method

**DOI:** 10.3390/ma9030153

**Published:** 2016-03-04

**Authors:** Yanghai Gui, Jianbo Zhao, Jingbo Chen, Yuanli Jiang

**Affiliations:** 1 School of Materials Science and Engineering, Zhengzhou University, Zhengzhou 450001, China; chenjb@zzu.edu.cn; 2 Henan Collaborative Innovation Center of Environmental Pollution Control and Ecological Restoration, Zhengzhou University of Light Industry, Zhengzhou 450002, China; zhaojianbo@zzuli.edu.cn; 3 Post-doctoral Research Base of Research Institute of Henan Energy and Chemical Industry Group Co.; Zhengzhou 450046, China; sensors741@gmail.com

**Keywords:** cBN, Ni/SiC, spines, electroplating, characterization

## Abstract

Cubic boron nitride (cBN) is widely applied in cutting and grinding tools. cBN grains plated by pure Ni and Ni/SiC composite were produced under the same conditions from an additive-free nickel Watts type bath. The processed electroplating products were characterized by the techniques of scanning electron microscopy (SEM), X-ray diffraction (XRD) and thermoanalysis (TG-DTA). Due to the presence of SiC particles, there are some additional nodules on the surface of Ni/SiC plated cBN compared with the pure Ni plated cBN. The unique morphology of Ni/SiC plated cBN should attain greater retention force in resin bond. Moreover, the coating weight of cBN grains could be controlled by regulating the plating time. cBN grains with 60% coating weight possess the optimum grinding performance due to their roughest and spiniest surface. In addition, Ni spines plated cBN grains show good thermal stability when temperature is lower than 464 °C. Therefore, the plated cBN grains are more stable and suitable for making resin bond abrasive tools below 225 °C. Finally, the formation mechanism of electroplating products is also discussed.

## 1. Introduction

It is well known that the superabrasive cBN has many excellent properties such as high thermal conductivity, high stability, superhardness, wide bandgap, good optical transparency in a wide range [1,2,3,4,5]. These merits make cBN the unrivaled material for the fabrication of cutting tools, grinding materials, super-hard protective coatings, optoelectronic devices, *etc.* Moreover, by contrast with diamond, cBN is chemically inert in machining ferrous materials and hence is a rather competitive material for mechanical applications. However, the practical applications of cBN are limited by its poor adhesion to the resin matrix, which results in detachment of cBN during the grinding process. In order to extend the lifetime of grinding tools, which can significantly improve production quality and lower production cost, much work has been done aiming to improve the adhesion of the abrasive grains. Coating metal or metal oxides on the surface of superhard abrasives is one of the effective methods to improve adhesion between cBN grains and resin bond [6,7,8]. At present, an effective way to improve the adhesion between cBN grains and resin matrix is to plate Ni metal on the surface of grains, which has been proved in practice application. But the conventional plating technique only makes a smooth Ni metal coating layer on the surface of cBN particles. It is believed that the electroplated cBN grains with fine rough and spiny morphology should contribute to enhancing the outstanding bond retention in resin matrix and saving the premature loss of grains from tools.

In the present study, electroplating pure Ni and Ni/SiC composite on the surface of cBN grains were investigated. Composite electroplating is a method of codepositing micron- or nano-sized solid particles [9,10,11,12]. These particles are usually two or more kinds of the hard oxides, nitrides, carbides, or even polymeric particles, such as Al_2_O_3_, SiO_2_, TiO_2_, SiC, WC or diamond, which can improve wear resistance, hardness of composite coatings or reduce friction. Yet, to our best of knowledge, little work has been reported to investigate Ni spines and nodules electroplated on cBN grains surface.

## 2. Materials and Methods

### 2.1. Sample Preparation

Pure Ni plated cBN grains and Ni/SiC plated cBN grains were electrolytically deposited in an additive-free nickel Watts type bath, respectively. The electroplate experiments were realized on a superhard materials barrel plating machine (JX41-5, Abrasives and Grinding Co. Ltd., Zhengzhou, China) with a rotation velocity of 3 rpm under direct current condition. The substrates that also used as cathode were cBN grains of 100/120 mesh (125–150 μm), which were processed through the following steps: oil removal, roughness, sensitization-activation, reduction and then chemical plating processing before electroplating [13]. Acid electroless plating bath was used in our work. Sodium hypophosphite was used as reducing agent. The electroless layer contained 13.09% P and an amorphous coating. The anode, which was a nickel foil of 99.9% purity with regular shape of 5 cm × 150 cm (wide and long), was positioned in the bath of the barrel plating machine. Before electroplating, the selected commercial SiC powders with a mean diameter of 5 μm were added to the bath. During electrodeposition, the temperature of the plating solution was maintained between 25 and 35 °C and the pH value of the electrolyte varied from 4.7 at the beginning of the deposition to 5.5 at the end. Plating current was kept between 1.0 and 1.5 A. Pure Ni plated cBN grains were also produced under the same experimental conditions for comparison. The composition of the plating solution and the deposition parameters for the preparation of plated cBN grains are presented in Table 1.

After plating, cBN grains were cleaned in distilled water for 10 min so as to remove loosely adsorbed SiC particles from their surface. Thus, plated cBN grains samples (S1, S2, S3 and S4) could be obtained after being dried in vacuum oven at 80 °C for 5 h.

### 2.2. Characterization

The microstructure was observed by scanning electron microscopy (SEM, JSM-6490, Jeol, Tokyo, Japan). The crystalline structure of plated cBN grains was examined by applying X-ray diffraction (XRD) technique utilizing a Bruker D8 diffractometer (Bruker-AXS, Karlsruhe, Germany) with a Cu-Kα radiation at 40 kV and 60 mA. The TG-DTA measurement was carried out with a ZRY-1 instrument (Jiangdong, Suzhou, China) at a heating rate of 15 °C/min with air as the buffer gas.

## 3. Results and Discussion

### 3.1. The Morphologies of Plated cBN Grains

**Figure 1:**
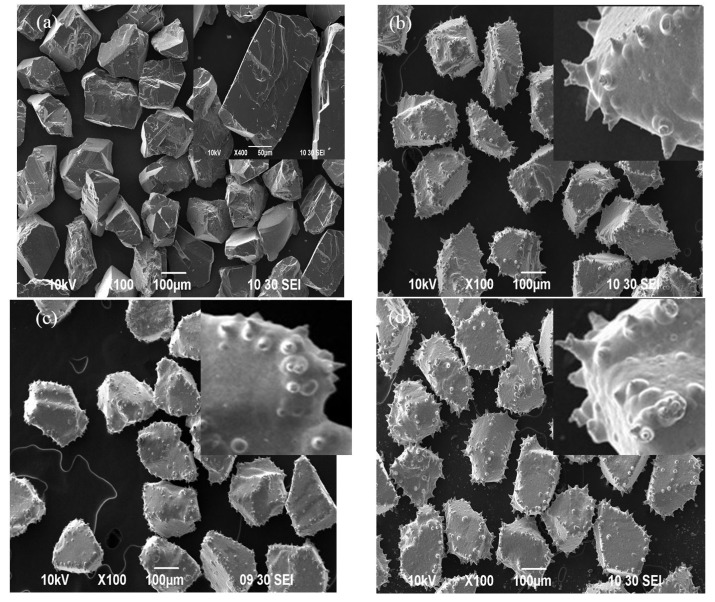

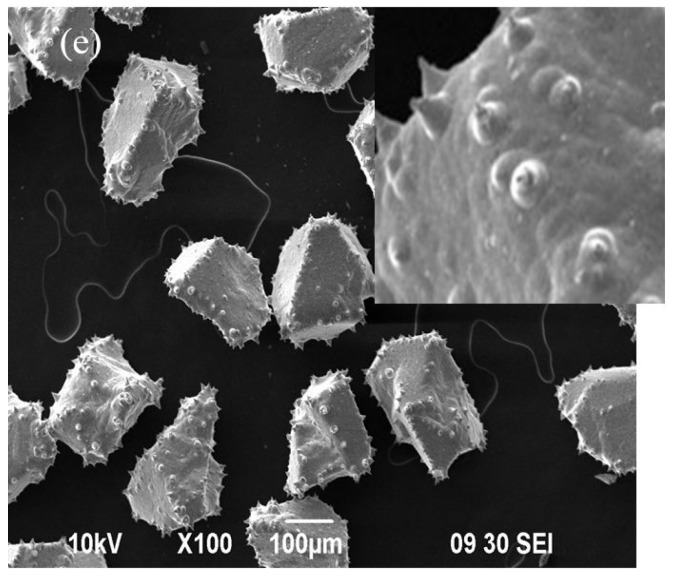
The SEM images of: (**a**) raw cBN grains; (**b**) S1; (**c**) S2; (**d**) S3; and (**e**) S4. Insets of **a**–**e** are their corresponding magnified images and S1, S2, S3, S4 are the samples referred to in Section 2.1 and Table 1.

The SEM images of raw cBN grains, pure Ni plated cBN and Ni/SiC plated cBN are shown in Figure 1. The original shape and surface texture of cBN grains (Figure 1a) cannot be seen due to the nickel coating after chemical plating, and the fine rough and spiny surface morphologies were also presented (Figure 1b–e). Very long and thick spines appear at the edge and bumpy surface of cBN matrix. When the plated cBN grains are used in resin bond tools, excellent bond retention and long tool life may be expected. In addition, there are some nodules on the surface of Ni/SiC plated cBN grains (Figure 1c–e), but none is on the surface of pure Ni plated cBN grains (Figure 1b). The unique morphology of the Ni/SiC plated cBN should give more outstanding adhesion in resin matrix and reduce the falling off of grains during grinding. Consequently, the wheels life would be greatly increased.

During electroplating process, the formation of Ni spines could result from two main aspects. On the one hand, the electric power line distribution was nonuniform on cBN grains surface, and the current density was higher at the edge and bumpy surface of the grains. On the other hand, there were a lot of crystal defects at the edge and bumpy surface of cBN grains where the surface free energy was higher. So nickel ions deposits could be easily produced at these places in order to reduce the surface energy, which should be responsible for the formation of Ni spines. In the case of Ni/SiC codeposition, adsorbed SiC particles at the edge and some bumpy surface of grains by an intra-crystalline mechanism resulted in the production of composite coatings with more structural defects than those of pure Ni coatings, and these defects may provide more growing points for nickel ion deposition. Thus, nickel particles in the defect place are rapidly deposited, resulting in some nodules that grew on cBN grain surface, as illustrated in Figure 1c–e. The morphology of plated cBN with Ni spines could also be affected by the concentration of SiC in bath. The Ni/SiC plated cBN grains take on the best morphology of Ni spines (Figure 1d) at the concentration of 10 g/L for the SiC additive. However, with the increase of SiC concentration in solution, no more nodules grew on cBN grains surface (Figure 1e). It may be that the surface of cBN grains covered with more adsorbed SiC particles made fewer activity centers of cBN grains surface.

### 3.2. The Weight Increment of Plated cBN Grains

The coating weight of pure Ni plated cBN and Ni/SiC plated cBN may be changed through the control of plating time. The longer the time, the thicker the coating. Metal nickel coating with moderate thickness can absorb and dissipate thermal-induced reaction during grinding, which resulted in the greatly improving of the grinding wheel life. However, a too thick metal coating will lower the grains friability and cutting efficiency, since the friable grains are necessary for the self-dressing ability of resin bond wheels [14]. In contrast, if the metal coating is too thin, Ni spines grow fine but small. It is difficult to attain sufficient retention force between cBN grains and the matrix. The coating weight may be calculated in two ways. One was evaluated based on deplating, and the other was based on the count values of the superhard materials barrel plating machine. After deplating, the coating weight of cBN grains may be about 60%. According to the calculation results of the barrel plating machine, the coating weight may be also approximately 60%. It should be noted that the two calculation results were well consistent with each other. Plated cBN grains with 60% weight increment exhibit very rough, spines morphologies (Figure 1d) that may improve outstanding adhesion in resin matrix. Therefore, the plated cBN grains should give optimum grinding performance.

### 3.3. The Phase Composition of Plated cBN Grains

**Figure 2:**
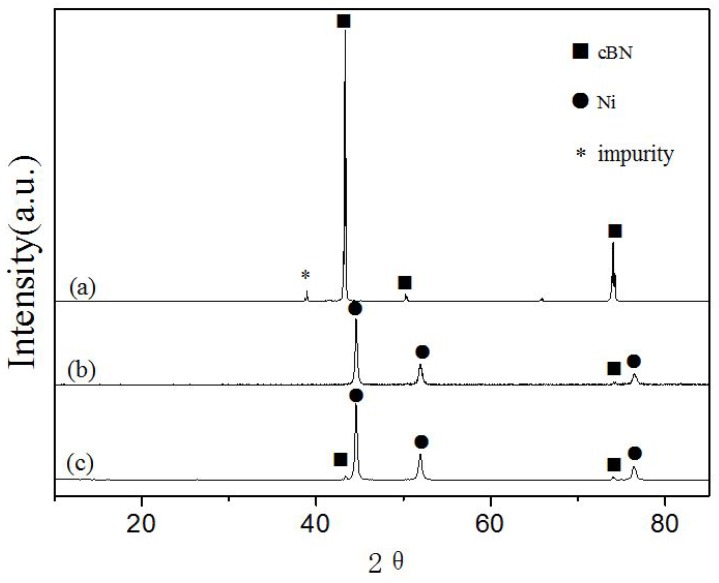
XRD patterns of: (**a**) raw cBN grains; (**b**) pure Ni plated cBN (S1); and (**c**) Ni/SiC plated cBN (S3).

The XRD patterns of raw cBN grains, pure Ni plated cBN grains and Ni/SiC plated cBN grains, prepared under the same conditions, are presented in Figure 2. The diffraction peaks are assigned to face-centered cubic cBN (JCPDS 25–1033) for raw cBN grains sample (Figure 2a). Another peak is ascribable to an unknown impurity, which should result from impurity trapped in cBN crystals during their synthetic process. After plating, all XRD peaks (Figure 2b) are assigned to face-centered cubic Ni (JCPDS 04–0850) and face-centered cubic cBN (JCPDS 25–1033) for plated cBN grains sample. The phases of cBN crystals and metallic Ni coexist in the XRD patterns, and the peaks of metallic Ni are very stronger than those of cBN crystals. This phenomenon indicates that plated products are cBN grains with core-shell structure. The metallic Ni shells content increased with the increase of electroplating time. As a result, the plating thickness also increased, resulting in the peaks of cBN crystals being covered up by those of metallic Ni. Any peak of the SiC diffraction peaks was not observed due to relatively small amount of SiC in the plated product.

### 3.4. Thermal Stability of Plated cBN Grains

**Figure 3:**
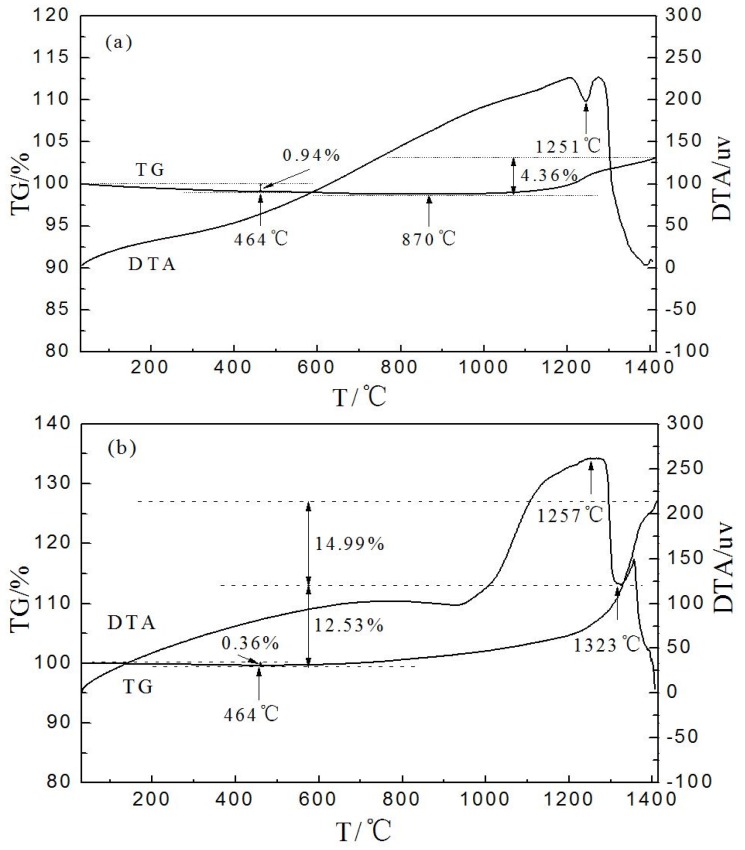
Thermoanalysis (TG-DTA) curves of: (**a**) raw cBN grains; and (**b**) Ni plated cBN.

TG-DTA curves of raw cBN grains and Ni plated cBN grains are presented in Figure 3a and b, respectively. Thermal data from TG (Figure 3a) indicates cBN grains possess high thermal stability and excellent oxidation resistance without significant weight loss till 870 °C in air atmosphere. The broad exothermal peak between 870 and 1417 °C and the corresponding mass increase of 4.36% should be due to the oxidation of cBN grains. There is a very weak endothermal peak center at around 1251 °C, which should be assigned to the melting of B_2_O_3_ derived from the oxidation products. The oxidation reaction of cBN grains described as follows [15]:

4BN + 3O_2_ = 2B_2_O_3_ + 2N_2_ (1)

There is a slight mass loss of 0.36% from 31 to 464 °C in the Ni plated cBN sample (Figure 3b). Yet the mass loss is about 0.94% at the same temperature in the TG curve of raw cBN grains (Figure 3a). The mass loss should be both ascribed to the desorption of substances physically and chemically absorbed on the surface of cBN grains. It is thus suggested that Ni plated cBN grains exhibit better thermal stability compared to raw cBN grains, which effectively protects the spines and nodules on the coatings surface from oxidization and thermal attacks during resin bond cBN wheels manufacturing and grinding. Therefore, the Ni plated cBN grains are more suitable for making resin bond abrasive tools below 225 °C. In the range of 464–1323 °C, there is a very strong exothermal peak at 1257 °C, accompanying a mass increase of about 12.53%. The exothermal peak and the corresponding mass increase should be assigned to the oxidation of nickel shells. There is a very weak exothermal peak at 1323–1412 °C, accompanying a mass increase of about 14.99%, which should be ascribed to the oxidation of cBN grains cores. In fact, the nickel shells usually changed prior to cBN grains cores in the oxidation process of cBN grains with core-shell structure. Based on the mass increase of 12.53% of nickel shells in 464–1323 °C, the weight increment of plated cBN grains is about 46%. It is suggested that the two exothermal peaks were partially superimposed at about 1323 °C. The oxidation of nickel shells may continue when the temperature is higher than 1323 °C.

### 3.5. The Forming Process of Ni Spines Plated cBN Grains

**Figure 4:**
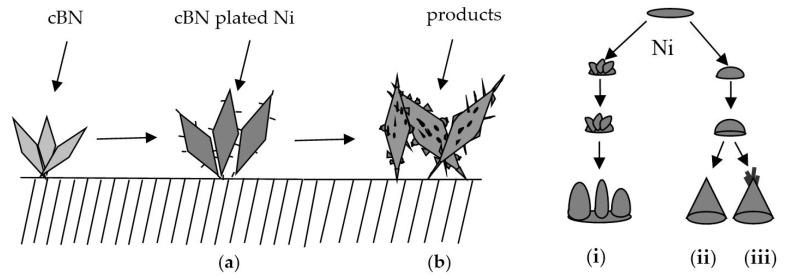
Schematic illustration of the forming processes of: (**a**) Ni spines plated cBN and (**b**) three Ni spines: (**i**) multiple spines, (**ii**) icicle spines and (**iii**) pronged spines.

Figure 4 shows the schematic representation of the forming process of Ni spines plated cBN grains. The process can be roughly bracketed into three periods: the early period, the middle period and the late period. In the early period, there was a thin layer of metallic nickel on cBN grains surface with the increase of plating time. In addition, some small Ni spines began to appear at the edge of cBN grains, as illustrated in Figure 4a. In the middle period, nickel ions unceasingly deposited on cBN grains surface, and at the same time some nodules appeared on the surface when the presence of SiC particles in bath. Adsorbed SiC particles on some surface of grains resulted in the production of composite coatings with more structural defects than those of pure Ni coatings, and these defects may provide more growing points, which was presumably responsible for the formation of nodules. Meanwhile, Ni spines grew longer and thicker than those of early period, and the deposition layer thickness also obviously increased when the plating time increased. In the late period, Ni spines got much longer and thicker, and nodules became even bigger, which should exhibit good retention in resin bond.

There are three shapes of Ni spines in electroplating products including icicle spines, pronged spines and multiple spines, as presented in Figure 4b. In the process of deposition, nickel ions more easily deposited the surface where nickel ions had already deposited. New nickel layer was closer to the anode. Therefore, the current on the nickel particles surface increased, and the cathode polarization also increased, which was more advantageous to form the new nickel nucleus, resulting in the formation of icicle spines. In the nickel nucleus growth process, cBN grains would collide with each other in the bath during the movement of cBN grains. So the tips of the original nickel nucleus may easily produce new interface defects, which were the new growing points where nickel ions started to discharge again. So, the multiple spines were soon formed. In the further growth process of the nickel nucleus, the tips of icicle spines became more and more thin. Thus, they collided more frequently in the bath, and new nickel layer formed again. The tips of Ni spines grew fine and small at the end of electroplating time. Therefore, pronged spines may be formed. Among the three shapes of Ni spines, pronged spines should have the greatest retention force in resin bond.

## 4. Conclusions

Pure Ni and Ni/SiC plated cBN grains were produced under the same conditions from an additive-free nickel Watts type bath, respectively. Plated cBN grains display very rough and spiny morphology, which should attain outstanding retention force in resin bond cBN wheels, and could reduce the falling off for the cBN grains during grinding. The longer life of tool would be expected. However, due to the presence of SiC particles, some nodules appear on the surface of Ni/SiC plated cBN grains compared to pure Ni plated cBN grains. The unique morphology of the Ni/SiC plated cBN grains should attain greater retention force in resin bond, which is most expected in practical application. Moreover, the coating weight of cBN grains may be changed. cBN grains with 60% coating weight should give optimum grinding performance. Plated cBN grains show good thermal stability below 464 °C, and effectively protect the spines and nodules from thermal attacks during use. Thus, the plated cBN grains are more suitable for making resin bond abrasive tools below 225 °C. Finally, the formation mechanism of Ni spines plated cBN is also proposed. There are three shapes of Ni spines in electroplating products including icicle spines, pronged spines and multiple spines. Among them, pronged spines should be the best one based on retention in the resin bond.

## Figures and Tables

**Table 1:** Overview of electrodeposition parameters for preparation of pure Ni plated cubic boron nitride (cBN) and Ni/SiC plated cBN.

Item	S1	S2	S3	S4
ρ(NiSO_4_·6H_2_O)/(g·L^−1^)	275	275	275	275
ρ(NiCl_2_·6H_2_O)/(g·L^−1^)	45	45	45	45
ρ(H_3_BO_3_)/(g·L^−1^)	37.5	37.5	37.5	37.5
ρ(SiC)/(g·L^−1^)	0	5	10	15
pH	4.7~5.5	4.7~5.5	4.7~5.5	4.7~5.5
Temperature/(°C)	25~35	25~35	25~35	25~35
Velocity/(rpm)	3	3	3	3
Current/(A)	1.0~1.5	1.0~1.5	1.0~1.5	1.0~1.5
